# Effects of 2-bromoethanesulfonate alone or in combination with calcium propionate or monensin on methane and gaseous hydrogen production during in vitro rumen fermentation

**DOI:** 10.3168/jdsc.2025-0795

**Published:** 2025-09-25

**Authors:** B. Rinn, A.K. Neff, G. Fincham, P.J. Kononoff, A.L. Carroll

**Affiliations:** 1 Department of Animal Science, University of Nebraska–Lincoln, Lincoln, NE 68504; 2 Department of Animal Sciences, Washington State University, Pullman, WA 99163

## Abstract

Some feed additives that mitigate enteric methane (CH_4_) production can result in an increase in hydrogen (H_2_) production. When expelled to the environment, H_2_ may intensify competition for hydroxyl radicals, prolonging CH_4_ persistence in the atmosphere. Therefore, the objective of the experiment was to determine the effects of adding 2-bromoethanesulfonate (BE) alone and in combination with monensin or calcium propionate on in vitro ruminal CH_4_ and H_2_ production. Using 3 separate runs, 1 g of 50% corn silage and 50% alfalfa (DM basis) was fermented (1) alone (control; CON); (2) CON with 100 μ*M* BE (BES); (3) BES + 2.5 μ*M* monensin (BM); and (4) BES + 2.5% DM calcium propionate (BC). Inoculum was obtained by collecting a mixture of rumen fluid from 2 steers (BW = 657 ± 12.5 kg) consuming a diet of 30% concentrate and 70% roughage. For each treatment within run, gas production was measured over a 48-h period using 2 randomly assigned paired and separate modules. Total CH_4_ and H_2_ production were calculated by multiplying the total volume of gas produced by the concentration of CH_4_ and H_2_ determined by gas chromatography at 0, 4, 8, 18, 24, and 48 h. Data were blocked by run and analyzed as a repeated measures randomized complete block design using the GLIMMIX procedure of SAS (v. 9.4) where treatment, time and the interaction between these 2 factors were considered as fixed effects and module, run and the interaction were included as random effects. Significance was declared at *P* ≤ 0.05. The BES, BM, and BC treatments did not differ in CH_4_ production (g/h) at 4 and 8 h of incubation, averaging 0.005 and 0.013 ± 0.0020 g/h, respectively, but CH_4_ production was reduced compared with the CON (0.017 and 0.047 ± 0.0018 g/h CH_4_, respectively). At 8 h, BES increased H_2_ production relative to BM and BC (0.002 and 0.0014 ± 0.0002 g/h H_2_, respectively), but all treatments containing BES were significantly greater than CON (0 vs. 0.002 ± 0.0002 H_2_ g/h). In conclusion, treatments containing BES reduced CH_4_, and the addition of calcium propionate or monensin may serve as a short-term strategy for the reduction of H_2_ resulting from mitigation of enteric CH_4_ production.

## Highlights

• 2-Bromoethanesulfonate (BE) decreased methane gas production in vitro.
• Monensin may increase CH_4_ mitigation effects when paired with BE.
• Monensin and calcium propionate may serve to reduce gaseous H_2_ production.

## Graphical Abstract

**Figure:**
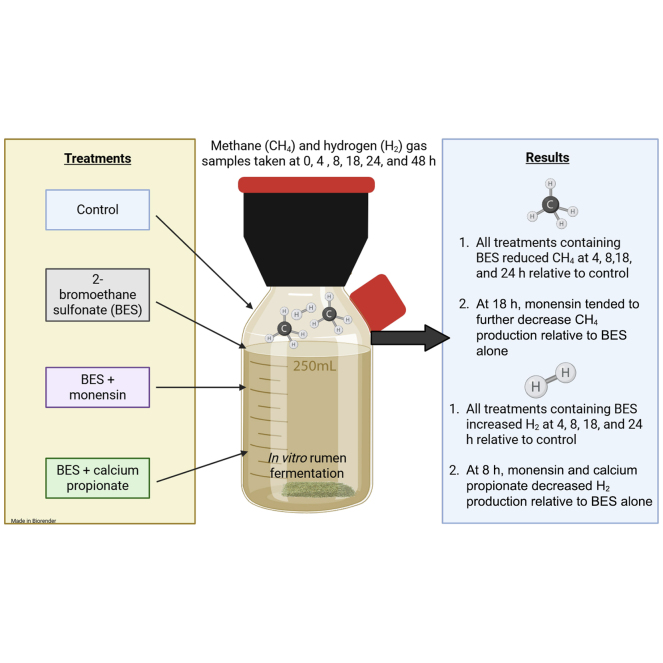
**Summary:** This experiment evaluated the effect of 2-bromoethanesulfonate in combination with monensin or calcium propionate on in vitro gaseous CH_4_ and H_2_ production. Gas pressure and composition data were collected at 0, 4, 8, 18, 24, and 48 hours of fermentation. Results suggest all treatments containing 2-bromoethanesulfonate reduced CH_4_ production, but monensin may further reduce CH_4_ compared with 18 hours of 2-bromoethanesulfonate alone. The addition of monensin and calcium also reduced H_2_, and this observation serves as evidence that the compounds may serve as a short-term strategy to reduce gaseous H_2_ production when CH_4_ mitigators such as 2-bromoethanesulfonate are used to reduce enteric CH_4_ production.

As the effects of climate change unfold, many sectors of the economy are responding to the call for strategies to reduce GHG emissions. Agriculture is one sector that is both a notable sufferer of and contributor to climate change spurred by GHG emissions. For example, in the case of dairy production, climate change poses an increased risk of heat stress in cows, which negatively affects milk production (St-Pierre et al., 2003; West, 2003); meanwhile, these dairy cows are also the world's second largest source of methane (CH_4_) emissions (Tricarico et al., 2022). One method to reduce CH_4_ production in dairy cattle is through feed additives that inhibit ruminal methanogenesis (Hristov, 2024). When the feed additive 3-nitrooxypropanol (**3-NOP**) is fed to cows, it directly inhibits enzymes utilized in methanogenesis, whereas other feed additives, such as monensin, alter the flow of H_2_ from methanogenesis to propionate production (Belanche et al., 2025). One other such product that is less frequently used in ruminants is 2-bromoethanesulfonate (**BE**), which has a similar mode of action to 3-NOP and is known to inhibit the ability of methyl-CoM reductase that catalyzes the final step of methanogenesis (Gräwert et al., 2014). Although it shows promise as a CH_4_ mitigator alone, there is little knowledge on how BE behaves in combination with other additives (Behlke, 2007). Although BE may act to reduce CH_4_ (Ungerfeld et al., 2004) it may also lead to an increase in hydrogen (H_2_; Lee et al., 2009), a response also observed with 3-NOP (van Gastelen et al., 2020). Should these phenomena hold, compounds that act as an acceptor for H_2_ could improve energy capture (Maigaard et al., 2024), and because H_2_ is an indirect GHG (Sand et al., 2023), also reduce the contributions of enteric GHG emissions. However, unlike 3-NOP, BES does not have a secondary mode of H_2_ utilization through the reduction of nitrate to nitrite, allowing for direct evaluation of potential H_2_ acceptors (Duin et al., 2016). The addition of calcium propionate and monensin, which leads to the increased production of propionic acid and propionate, a known alternate pathway for H, could serve as an acceptor for H_2_ (Pereira et al., 2022). The objective of this in vitro experiment was to explore the effects of BE on rumen CH_4_ production and determine if the addition of calcium propionate or monensin reduces gaseous H_2_ production. We hypothesized that monensin and calcium propionate would reduce gaseous H_2_ production when the CH_4_ mitigator BE was added.

In this study, gas production was measured in vitro using the ANKOM RF Gas Production System (Ankom Technology, Macedon, NY). We used 16 individual 250-mL bottles equipped with individual gas production modules used to measure gas pressure. Using 3 separate runs, a 1-g mixture consisting of 50% corn silage (96.5% DM, 8.3% CP, 38.8% NDF, 34.7% starch, and 3.7% ether extract) and 50% alfalfa (96.2% DM, 19.4% CP, 42.1% NDF, 1.4% starch, and 2.0% ether extract) was fermented (1) alone (control; **CON**); (2) CON with 100 μ*M* BE (**BES**; Behlke, 2007); (3) BES + 2.5 μ*M* monensin (**BM**; Karnati et al., 2009); and (4) BES + 2.5% DM calcium propionate (**BC**). The 2.5% diet DM calcium propionate dosage represents the upper amount provided to lactating dairy cattle experimentally (Stokes and Goff, 2001). Within each run, 1 of 4 treatments were assigned to each of 16 fermentors, and fermentors were randomly assigned as pairs within treatment with one serving as the fermentor from which gas was sampled and the second used to measure gas pressure.

All animal care and experimental procedures were approved by the University of Nebraska–Lincoln Animal Care and Use Committee (#2226). Inoculum was obtained by collecting a mixture of rumen fluid from 2 steers (BW = 657 ± 12.5 kg) fed twice daily (0700 h and 1300 h) a TMR composed of 30% concentrate and 70% roughage (Table 1). The steers were housed in individual pens in a temperature-controlled (20°C) barn with continuous access to water at the Beef Metabolism facility located at the University of Nebraska–Lincoln. At 1200 h, 2 L of rumen contents were collected from each steer, and this fluid was strained through 4 layers of cheesecloth (IDEALFOLD Cheesecloth, Grainger, Omaha, NE) and placed into prewarmed insulated containers (Thermos LLC, Schaumburg, IL). Whole rumen contents were placed into separatory funnels (Thermo Fisher Scientific Inc., Waltham, MA) and purged with CO_2_, after which a stopper was placed on the top, and the entire funnel was placed in a 39°C water bath for ∼15 min. The less dense liquid portion was separated from the ruminal contents and added in a 1:1 ratio to reduced and prewarmed (39°C) McDougall's buffer (1 g urea/L). Next, 100 mL of the McDougall's buffer inoculum mixture was added into each of the fermentors containing the treatment. Fermentors were then immediately purged with CO_2_ gas for 5 s to ensure anaerobic conditions, and their respective modules were tightly secured and then placed in a 39°C water bath (Precision Shaking Water Bath SWB 27, Thermo Fisher Scientific Inc., Waltham, MA).

**Table 1:** Ingredient inclusion and chemical composition of experimental diets for rumen fluid donor steers^1^

Item	% of diet DM
Ingredient	
Grass hay	69.8
Corn dried distillers grains	23.5
Corn grain, dry rolled	5.94
Salt	0.287
Vitamin ADE premix	0.030
Trace mineral mix	0.048
Limestone	0.377
% Diet DM	
CP	16.7
NDF	53.8
Starch	5.65
Ether extract	3.09
Ash	10.2

1 Red Angus steers (n = 2) averaging 15.1 kg/d DMI.

Gas production was measured in each fermentor over 48 h. Fermentors were set to release pressure at 2 kPa, and pressure data were recorded every 5 min for 48 h. An SRI 8610C gas chromatograph (SRI Instruments, Torrance, CA) was used to measure gas composition. At 0, 4, 8, 18, 24, and 48 h, 6 mL of gas was manually extracted from the 8 sampling fermentors via the septum port of the bottles using a 25-mL gas-tight syringe (Hamilton Co., Reno, Nevada), and the gas was then injected into the GC for CH_4_ analysis. The GC was programmed to measure the concentration of CH_4_ in 2-min intervals, and helium was used as a carrier gas. The GC was calibrated using a certified gas containing 0.08723% CH_4_, 0.8828% carbon dioxide, 20.0209% oxygen, and 79.00907% nitrogen. For H_2_ analysis, two 6-mL gas samples were extracted from the fermentor and placed into a gas-tight prepurged 12-mL glass breath testing vial (ALWSCI Technologies, Shaoxing, Zhejiang, PR China). Before sampling, each 12-mL gas-tight glass breath testing vial was purged with N_2_ gas, and 6 mL of the gas (50%) was extracted from the vial with a 25-mL gas-tight syringe (Hamilton Co., Reno, NV). Vials were collected in duplicate from each fermentor per time point, and during each time point, 4 vials served as blanks to correct in case of H_2_ infiltration. Vials were then analyzed for H_2_ using a Trace Analytical RGA3 reduced gas analyzer (Ametek Process Instruments, Newark, DE) calibrated with serial dilutions of a 1,000 mg/kg H_2_ standard (Sigma-Aldrich, St. Louis, MO). Total gas production was calculated using the ideal gas law (PV = nRT), where n = gas produced in moles (mol), P = pressure in kilopascals (kPa), V = headspace volume in glass fermentation bottle in liters (L), T = temperature in Kelvin (K), and R = gas constant (8.314472 L·kPa·K^−1^ ·mol^−1^). Using Avogadro's law, 1 mol of gas at atmospheric pressure measured in psi (1 psi = 6.894757293 kPa) will occupy 22.4 L at 273.15K and 101.325 kPa. The gas, which was measured in moles, was then converted to grams by multiplying by the respective molar masses of 16.04 g/mol and 2.016 g/mol for CH_4_ and H_2_, respectively.

Three separate runs were conducted, and data were blocked by run and analyzed as repeated measures in a randomized complete block design using the GLIMMIX procedure of SAS (v. 9.4, SAS Institute). To do so, treatment, time, and the interaction between these 2 factors were considered as fixed effects, and module pair, run, and the interaction were included as random effects. All covariance structures were fitted, but autoregressive 1 was chosen based on the lowest Bayesian information criterion. Statistical significance for all treatments effects was declared at *P* ≤ 0.05, and trends discussed at *P* > 0.05 and ≤0.10.

The objective of the current experiment was to quantify CH_4_ and H_2_ production when adding BES alone or in combination with monensin and calcium propionate. We observed that BES alone reduced CH_4_ production by 52%, which was likely due to inhibition of methyl coenzyme M reductase (Behlke, 2007). Methyl coenzyme M reductase serves as the final step within the Wolfe cycle of methanogenesis, and BES, like 3-NOP, would serve to inactivate methyl coenzyme M reductase through the oxidation of the Ni atom (Belanche et al., 2025). More specifically, compared with CON, the treatment containing BES reduced (*P* < 0.01: Figure 1A) CH_4_ production (g/h) at 4, 8, and 18 h of fermentation (0.017 vs. 0.005 ± 0.0020, 0.047 vs. 0.013 ± 0.0018, and 0.033 vs. 0.019 ± 0.0018 g/h CH_4_ for CON vs. BES treatment, respectively). The greatest reduction in CH_4_ production occurred at 8 h, with BES decreasing CH_4_ by 72% compared with CON. The compound BES is structurally analogous to methyl coenzyme M which catalyzes the final step of the Wolfe cycle (Duin et al., 2016). Other structural coanalogues of the methyl coenzyme M, most notably 3-NOP, have been used to reduce CH_4_ production in ruminants by ∼30% (Kebreab et al., 2023). Interestingly, compared with CON and BES at 18 h during peak gas production, the BM treatment tended to further decrease the rate (*P* ≤ 0.07; 0.033, 0.019, and 0.016 ± 0.0018 g/h, respectively) of CH_4_ production by 52% and 16%, respectively. Monensin has long been known to reduce enteric CH_4_ production in ruminants by as much as 5% (Ranga Niroshan Appuhamy et al., 2013). This is thought to occur because ionophores such as monensin selectively promote gram-negative bacteria that reduce succinate to propionate through the reduction of gram-positive bacteria (McGuffey et al., 2001). Propionate production utilizes H_2_ and is considered an alternative H_2_ pathway to CH_4_ (Belanche et al., 2025). Although the shift in H_2_ utilization for propionate production is a potential mode of action, VFA were not evaluated in the current experiment, and the possibility of this effect occurring should be further evaluated. Thus, consistent reduction in CH_4_ observed in all treatments containing BES indicates BES could serve as a useful mitigator for enteric CH_4_ production in ruminants. However, further detailed research with feed digestion, dissolved H_2_, and VFA production is required to fully elucidate if BES and monensin have synergistic CH_4_-mitigating effects.

**Figure 1:**
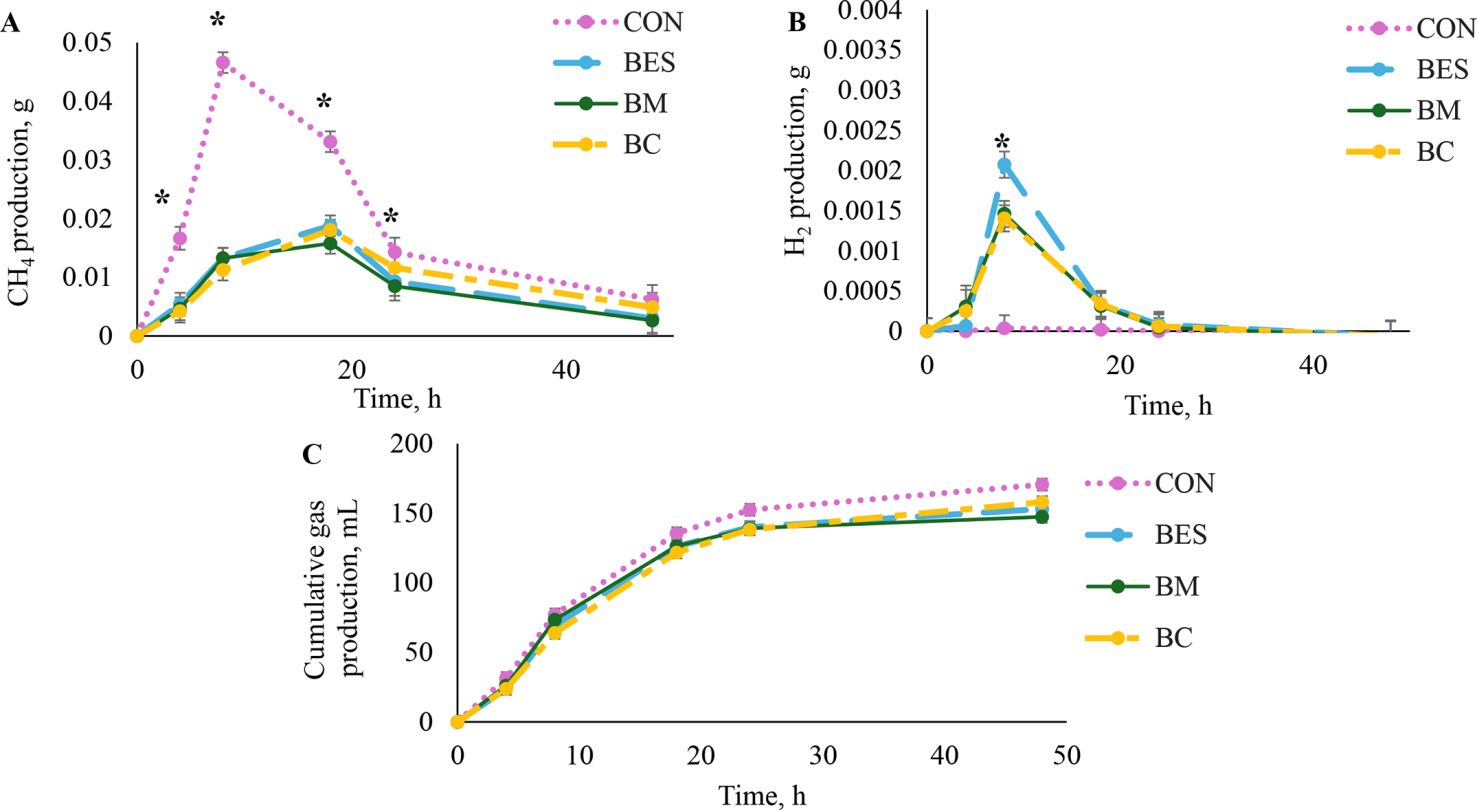
Gaseous CH_4_ production (g/h; A), gaseous H_2_ (g/h; B), and cumulative gas production (mL; C) during a 48-h period for 2-bromoethanesulfonate alone and in combination with monensin and calcium propionate. Treatment by time interactions were detected (A, B; *P* < 0.01), and a tendency for a treatment by time interaction was detected (C; *P* = 0.06). *Indicates significant treatment differences within time point. Treatments were (1) control (CON); (2) CON + with 100 μ*M* BE (BES); (3) BES + 2.5 μ*M* monensin (BM); and (4) BES + 2.5% DM calcium propionate (BC). Error bars are SE.

Gaseous H_2_ production can compete with hydroxyl radicals in the atmosphere, increasing the persistence of atmospheric CH_4_ (Bertagni et al., 2022). Strategies to decrease enteric CH_4_ production may increase H_2_ production, as CH_4_ is a primary source of H_2_ utilization in the rumen (Morgavi et al., 2010; Mitsumori et al., 2012; Ungerfeld, 2020). In the current experiment, we observed a short-term decrease in H_2_ production when combining BES with either monensin or calcium propionate. We speculate that this is because available H is used for propionate and propionic acid production. This notion is supported by our observation on gaseous H_2_ production (Figure 1B). Specifically, we observed that at 8 h of fermentation, BM and BC reduced (*P* ≤ 0.01) the H_2_ gas production rate by ∼31% compared with BES alone, but all treatments containing BES were significantly greater than CON (0 vs. 0.002 ± 0.0002 H_2_ g/h). However, the effect was short lived, and no difference (*P* > 0.88) was observed among treatments containing BES at 18 h or beyond. This effect was also not mediated by differences in total gas production (mL) between BES treatments (*P* ≥ 0.35; Figure 1C). As previously stated, monensin acts to increase respiratory hydrogenotrophs redirecting metabolic H_2_ flow toward propionate production (Badhan et al., 2025). Previous research has observed tendencies for decreased total H_2_ emissions in cattle fed 3-NOP + monensin relative to those fed 3-NOP alone in the 24 h subsequent to feeding (Vyas et al., 2018). The mechanism for reduction of gaseous H_2_ in the BC treatment is not clear. In the current study, pH was not measured, but when pH is below 6.0, VFA become more undissociated, which may lead to the conversion of calcium propionate and ^2^H^+^ to propionic acid (Dijkstra et al., 1993). To clarify, this mechanism would not directly utilize H_2_ produced from redox reactions available for methanogenesis, but H^+^ utilized in acid-base reactions (Tedeschi and Nagaraja, 2025). Thus, removal of H^+^ before reduction to H_2_ may result in decreased gaseous H_2_ in BC relative to BES. As a result, data indicate short-term reductions in H_2_ may be achieved with monensin and calcium propionate during CH_4_ inhibition with BES. Further research should explore the dynamic changes in H_2_ and quantify H_2_ transfers, as well as the potential of rumen microbes in developing resistance to the compounds tested.

The aim of this experiment was to quantify CH_4_ and gaseous H_2_ production when combining BES alone and in combination with monensin or calcium propionate. Results of the experiment show BES decreases CH_4_ production, and that monensin and calcium propionate may serve to further decrease gaseous H_2_ when mitigators such as BES are fed. Further research should continue to examine combinations of gaseous CH_4_ and H_2_ feed additives while concurrently evaluating responses in vivo. We suggest that key responses to be tested in vivo should include rumen VFA production, gaseous CH_4_ and H_2_, dissolved H_2_, and nutrient digestibility. Thus, direct improvements in enteric CH_4_ and H_2_ mitigation in ruminants may occur by understanding complimentary mechanisms of enteric CH_4_- and H_2_-mitigating feed additives both in vitro and in vivo, and characterizing the roles of H^+^ and H_2_ in enteric CH_4_ and H_2_ mitigation.
